# HBV and HCV Therapy

**DOI:** 10.3390/v1030484

**Published:** 2009-10-22

**Authors:** Pietro Lampertico, Alessio Aghemo, Mauro Viganò, Massimo Colombo

**Affiliations:** “A.M. Migliavacca” Center for Liver Disease, First Gastroenterology Unit, Fondazione IRCCS Maggiore Hospital, Mangiagalli e Regina Elena, Università di Milano, Via F. Sforza 35, 20122 Milan, Italy

**Keywords:** HBV DNA, nucleos(t)ide analogues, Peg-IFN, resistance, HCV RNA, Ribavirin, SVR

## Abstract

One year of interferon therapy inhibits HBV replication in one third of the patients whereas long-term administration of oral nucleos(t)ide analogues is efficient in most of them, as long as early treatment adaptation in patients with partial virological response and resistance is provided. Following the demonstration of a more potent antiviral effect in terms of sustained virological response (SVR) rates, Pegylated-IFN coupled with Ribavirin has become the standard treatment for chronic hepatitis C, with nearly 65% of all treated patients achieving a SVR. Long-term suppression of HBV and eradication of HCV would halt the progression of chronic hepatitis to cirrhosis, hepatocellular carcinoma and liver decompensation.

## 1. Treatment of Chronic Hepatitis B

Chronic hepatitis B viral infection affects about 400 million people around the globe, being one of the most common infectious diseases and among the world’s leading causes of death. Antiviral therapy of chronic hepatitis B (CHB) aims to improve quality of life and survival chance of the patients by preventing progression of liver damage to cirrhosis, end-stage liver disease and liver cancer (HCC), thus preventing anticipated liver-related death. This goal is achieved by suppression of HBV replication in a sustained or maintained manner, either by short-term “curative” treatment with standard (IFN) and pegylated interferon (Peg-IFN) or long-term “suppressive” therapy with nucleos(t)ide analogues, like lamivudine, adefovir, entecavir, telbivudine and tenofovir. Since both strategies have advantages and disadvantages, the wise treatment of a patient with CHB requires careful balance between prediction of the natural history of HBV and of the potential benefit of anti-HBV therapy. Recent data on the long-term efficacy of third generation of nucleos(t)ide analogues entecavir and tenofovir have tipped the balance towards long-term suppression therapy as the first-line option for most patients with CHB, independent of the HBeAg status.

### 1.1. Treatment Indications

Patients with clinical or histological predictors of HBV progression should be prioritized to anti-HBV treatment [1–3]. HBeAg seropositive immunotolerant patients with persistently normal alaninoaminotransferase (ALT) levels, high serum HBV DNA, minimal histological changes in the liver and no family history of HCC or cirrhosis, do not require immediate therapy. Inactive HBV carriers, *i.e.* HBsAg/anti-HBe seropositive patients with persistently normal ALT levels and less than 2000 IU/ml of serum HBV DNA, do not require therapy, as well as patients with slightly elevated ALT (less than 2 times the upper limit of normal) and histological hepatitis less than grade A2 or stage F2 by METAVIR scoring.

According to the recent EASL Practice Guidelines [3], both HBeAg-positive and HBeAg-negative patients should be considered for treatment when HBV DNA levels exceed 2000 IU/ml and/or the serum ALT levels are above the upper limit of normal (ULN), and a liver biopsy shows moderate to severe active necro-inflammation and/or fibrosis, *i.e.* grade A2 or stage F2 by METAVIR scoring. While indications for treatment must also take into account age, health status, and availability of anti-viral agents in individual countries, current guidelines support also the view that all patients with compensated or decompensated cirrhosis and detectable serum HBV DNA (even <2000 IU/ml) must receive antiviral treatment regardless of ALT. The main goal of treatment in these severely ill patients is to completely inhibit viral replication in order to improve liver function and survival and, in case of liver transplantation, to prevent graft re-infection.

### 1.2. End-points of Therapy

The treatment paradigm of CHB is to persistently suppress serum HBV DNA to as low a level as possible. This goal can be obtained either on- or off-treatment, with subsequent biochemical remission, histological improvement, prevention of complications and improved long-term outcomes. Maintenance of undetectable levels of HBV DNA is mandatory to prevent resistance to NUCs and increase the rates of anti-HBe and HBsAg seroconversion. In both HBeAg-positive and HBeAg-negative patients, the ideal end-point of therapy is sustained HBsAg loss with seroconversion to anti-HBs [4–6]. In HBeAg-positive patients, a sustained HBeAg seroconversion is a realistic and satisfactory end-point associated with improved prognosis [7–9]. In HBeAg-positive patients unable to seroconvert to anti-HBe and in HBeAg-negative patients, a maintained undetectable HBV DNA level on treatment with NUCs or a sustained undetectable HBV DNA level after IFN is the next most desirable end-point, as the induction of persistent biochemical and virological remission appears to be the most important therapeutic target in CHB [10–16].

### 1.3. Therapeutic Strategies

Currently, there are seven drugs licensed for treatment of CHB: standard interferon (IFNα), pegylated IFNα (Peg-IFNα), lamivudine (LMV), adefovir dipivoxil (ADV), entecavir (ETV), telbivudine (LdT) and tenofovir disoproxil fumarate (TDF). IFN couples antiviral and immunomodulatory activities and is administered subcutaneously, whereas the oral antiviral agents which are analogs of natural nucleosides (LMV, ETV, LdT) or nucleotides (ADV, TDF) are administered once daily. Two different therapeutic approaches can be used: short-term finite curative treatment based upon IFN administration and long-term suppressive treatment based upon NUC therapy.

#### 1.3.1. HBeAg-positive Chronic Hepatitis B

**Figure 1.**
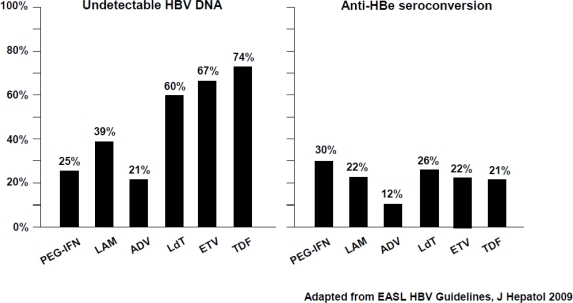
48-week rates of virological response (HBV DNA < 300/400 copies/ml) and HBeAg seroconversion in NUC-naïve HBeAg-positive patients. Collation of currently available data – not from head-to-head studies.

Treatment with IFN: In a minority of HBeAg-positive CHB, standard IFNα for 4–6 months or Peg-IFNα for 12 months can induce sustained off-treatment response. Following the administration of 5 million units (MU) daily or 10 MU thrice weekly of standard IFN or 180 μg Peg-IFNα-2a or 100 μg Peg-IFNα-2b per week, 25% of the HBeAg positive patients have had virological response in terms of HBeAg seroconversion, while HBsAg seroconversion occurred in 3% of the patients 6 months off-treatment [17–19] (Figure 1). Most patients may have a sustained treatment response, in association with a high likelihood of HBsAg loss [9]. After a 3-year post treatment follow-up, 37% and 11% of the patients who received Peg-IFNα-2b for 1 year became HBeAg and HBsAg seronegative, respectively, and among the initial responders, HBeAg and HBsAg were lost in 81% and 30% of the patients, respectively [20]. HBe seroconversion was more likely to occur in patients with high ALT (above 3 times ULN), low HBV DNA (below 7 log IU/ml) and high activity scores (≥ A2) on liver biopsy [17]. An HBV DNA decrease to less than 20,000 IU/ml at week 12 of IFN treatment is associated with a 50% chance of HBeAg seroconversion [21]. Patients with genotype A and B have a good end of treatment and off- treatment responses to IFN, with HBeAg loss occurring in one third of the patients, in addition to HBsAg seroconversion in 13%–22% of genotype A patients [22,23].

Additional therapy with NUCs will cause a more robust decline of HBV DNA levels without increasing the chances of sustained virological responses to IFN [18,19]. Despite increased risks of side effects and contraindications, a 48-week course of Peg-IFNα is a recommended as a first-line therapy for young, highly motivated, non-cirrhotic HBeAg-positive patients with genotype A or B having high pre-treatment serum levels of ALT [3].

Treatment with NUCs: NUCs are potent antiviral agents affecting the reverse transcription step of HBV replication. Finite duration treatment with NUCs is achievable for HBeAg-positive patients with undetectable HBV DNA (<10–15 IU/ml) and HBeAg seroconversion on treatment. The duration of therapy, however, is unpredictable depending on timing of HBeAg seroconversion. Patients lacking HBeAg seroconversion require long-term treatment with NUCs. Despite being well tolerated, long-term administration of NUCs is hampered by the selection of drug resistant mutants, leading to loss of efficacy, frequent relapse after discontinuation and by concern on long-term safety. Although anti-HBV potency of various NUCs may not be directly comparable because of the heterogeneity of assays used in the registration trials, substantial differences in the potency of antiviral agents in reducing serum HBV DNA levels have surfaced. Notwithstanding, differences in anti-HBV activity do not translate into different rates of HBeAg seroconversion, at least within the first year of therapy. The virological response rates at year one were 36%–40%, 21%, 67%, 60% and 74% with lamivudine, adefovir, entecavir, telbivudine and tenofovir, respectively, while the HBe seroconversion rates were approximately 20% for all NUCs (Figure 1) [24–29].

HBeAg seroconversion increases steadily with prolonged LMV treatment, reaching 27%, 40%, 47% and 50% at year 2, 3, 4 and 5, respectively [30,31] but long-term LMV monotherapy inexorably ends with the selection of resistant strains harbouring specific mutations in the HBV polymerase gene, *i.e.* M204I/V as a primary mutation and L80, L180M, V173L as the most relevant secondary mutations [32–35], at rates that increase from 20% after 1 year to peaks of 70% after 5 years of drug therapy [31,36]. Non-Asian ethnicity, high pre-treatment serum HBV DNA level, male sex, and high body mass index are well recognized predictors of LMV resistance [37]. Since the risk of developing LMV-resistance directly correlates with a slow response of serum HBV DNA at month 6, incomplete suppression of HBV replication seems to play a key role in the generation of mutated strains [38,39]. Following LMV therapy, the durability of HBeAg seroconversion appears to be smaller when compared to IFN, being 77% at 3 years in non-Asian patients and less than 60% at 2 years in Asian patients, however following shorter courses of therapy [40–42]. To overcome HBeAg seroreversion, LMV should be continued probably for an additional year. Prolonged LMV therapy may reduce the risk of disease progression and development of HCC [43], except in patients developing LMV-resistance. Indeed, the beneficial effects of LMV appear to be offset by the development of LMV-resistance to the point that recent current guidelines do not recommend LMV use anymore [3]. Prolonged therapy with ADV resulted in an increase of HBeAg seroconversion rates to 29% and 43% at year 2 and 3 [44], with a marginal genotypic resistance of 6%. Durability of HBeAg seroconversion may be as high as 90% in the absence of any serious adverse event [44]. The anti-HBV activity of ETV monotherapy results in a continuous viral decline beyond week 48, similarly to the rates of HBeAg seroconversion [45,46], in the presence of limited rates (1.2% at year 5) of resistance in NUC-naïve patients [47]. The same observation holds true for TDF: two-year treatment achieved HBV DNA < 400 copies/ml in 78% of patients with 30% and 6% of HBeAg and HBsAg loss, respectively [48]. Even though the antiviral potency expressed by the reduction in serum HBV DNA levels differs among the various anti-HBV agents (highest with TDF, ETV and LdT, intermediate with LMV and lowest with IFNα/Peg-IFNα and ADV), these differences do not translate into different HBeAg seroconversion rates, at least within the first year of therapy. HBsAg loss is < 2% after one year of treatment with NUCs, mimicking the natural history of the infection in the untreated patients.

Pre-treatment factors, predictive of HBeAg seroconversion following NUCs, included low viral load (HBV DNA below 7 log_10_ IU/ml), high serum ALT levels (above 3 times ULN) and high activity scores at liver biopsy (at least A2) [49]. During treatment with LMV, ADV or LdT, an undetectable HBV DNA by a real-time PCR assay at 24 or 48 weeks is associated with a reduced risk of resistance and an improved chance of a HBeAg seroconversion, without any influence of the HBV genotype [24,39].

#### 1.3.2. HBeAg-negative Chronic Hepatitis B

Treatment with IFN: Twelve to 24-month courses with IFNα (3 or 5 MU thrice weekly) resulted in sustained long-term off therapy responses in less than one third of patients who often (>40%) cleared HBsAg (10–12,50]. As sustained responses increased with treatment duration, HBsAg went lost in up to 67% of patients under long term follow-up [12]. In a large multinational trial with Peg-IFNα-2a ± LMV *versus* LMV monotherapy for 48 weeks, a biochemical and virological response was achieved in 35% of the patients treated with Peg-IFN [51]. Although combination of Peg-IFNα-2a and LMV led to higher end of treatment response rates than either drug alone, this effect went lost 6 months after treatment cessation. Viral response to IFN therapy was predicted by high baseline ALT and low HBV DNA levels, female gender, younger age and HBV genotype [52]. Sustained off-therapy response, however, tapered down during follow-up, with <10,000 copies/ml HBV DNA being detected in a quarter of patients treated with Peg-IFNα-2a ± LMV, while 9% of the patients in both groups lost HBsAg [53]. According to EASL Practice Guidelines, Peg-IFN is indicated to treat HBeAg-negative patients with the best chance of a sustained off treatment response [3].

**Figure 2.**
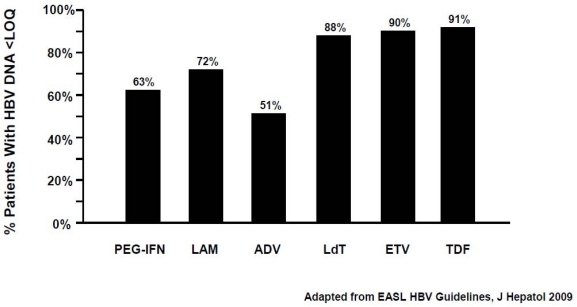
48-week rates of virological response (HBV DNA < 300/400 copies/ml) in NUC-naïve HBeAg-negative patients. Collation of currently available data – not from head-to-head studies.

Treatment with NUC: Short-term treatment with NUCs results in high on-therapy rates of virological responses in HBeAg-negative CHB: 72%, 51%, 90%, 88% and 91% with lamivudine, adefovir, entecavir, telbivudine and tenofovir, respectively, however with a high risk of relapse after treatment discontinuation, indicating the need for continuous administration of these agents [54–63] (Figure 2).

**Figure 3.**
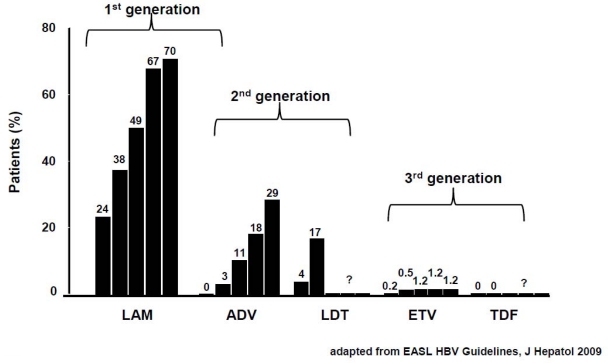
Incidence of resistance in NUC-naïve patients treated up to 5 years. Collation of currently available data – not from head-to-head studies.

In patients achieving viral remission, disease prognosis is overall favourably affected, even though development of viral resistance may jeopardize long term outcome of hepatitis due to virological and biochemical breakthrough, requiring rescue treatment [3,15,16] (Figure 3).

According to recent current guidelines, LMV monotherapy is no longer the recommended first-line strategy of therapy [3]. ADV monotherapy is no longer considered a first-line therapy either, since the proportion of patients with HBV DNA <1000 copies/ml and normal ALT levels is 67% and 69%, respectively, at year 5 of therapy [58]. In the face of few patients (5%) with a HBsAg loss, ADV monotherapy faces the risk of genotypic resistance, virological resistance (defined as >1 log rebound compared to on-treatment nadir) and clinical resistance (defined as virological and biochemical rebounds), which are 29%, 20% and 11%. Drug withdrawal in patients with persistent undetectable HBV DNA by PCR assays results in a virological relapse occurrence in all patients, although approximately 70% of them could maintain serum levels of HBV DNA below 10,000 copies and ALT levels within the normal range for at least 12–18 months [59]. Most of the few patients evaluated for 2 or 3 years on ETV treatment, had maintained virological response [60,61]. Despite the lack of long-term resistance data in HBeAg negative patients, the very low rates of resistance in HBeAg positive partial responders indicate that ETV monotherapy suppresses viral replication in most HBeAg negative patients for years [47]. In a 2-year study of LdT 600 mg/day, 82% of the nucleoside-naïve HBeAg-negative patients achieved undetectable HBV DNA by PCR assay [62], with genotypic resistance to LdT developing in 9% of the patients. A 24-week virologic response (HBV DNA < 2 or 3 log copies/ml) correlated with high rates of maintained PCR undetectability and negligible risk of LdT resistance after 2 years of therapy [63]. This data provides the rationale for adapting and tailoring antiviral therapy in 24-week suboptimal responders. The nucleotide analog TDF is a powerful anti-HBV drug, as shown by the 2-year study in which 91% of patients achieved HBV DNA < 400 copies/ml and most normalized ALT levels [64].

In HBeAg-negative patients, combination of two NUCs, as well as the use of Peg-IFN plus NUC, have so far not proven to be beneficial for providing better treatment outcome, however, combination as *de novo* treatment may prevent resistance. Long-term treatment with the most potent drugs with the optimal resistance profile, *i.e.* TDF or ETV, should be used as first-line monotherapy in HBeAg-negative patients who cannot benefit from interferon [3]. However, the long-term effects, safety and tolerability of ETV and TDF are still unknown.

### 1.4. Management of Treatment Failure

Working against long-term therapy with NUCs is the risk of the emergence of drug resistant HBV strains, ultimately leading to treatment failure and liver disease progression (Figure 3). Being the drug for rescuing a response to therapy selected on the basis of its *in vitro* cross resistance profile, NUC like LdT and ETV are unfit for treating LMV-R, since they share a similar resistance profile, characterized by the mutation in position 204 plus several compensatory mutations [65]. By converse, being the resistance pattern of the nucleotide analog ADV based upon changes at position 236 and 181, *i.e.* N23T and A181V, this drug is an option for treating LMV-R strains [66–68]. As a general rule, a nucleotide analog is recommended to rescue for HBV resistance in patients resisting to nucleoside analogues, whereas a nucleoside analog is recommended for a nucleotide-related resistance. EASL Practice Guidelines recommend TDF as the nucleotide of first choice for the rescue of nucleoside resistant strains of HBV [3]. Following selection of a rescue drug, the next step is to define whether to treat the patients with a monotherapy, *i.e.* by switching from LMV to ADV or TDF or other drugs, or with a combination therapy (“add-on” strategy). Both approaches have pros and contras: the former strategy is cheaper but potentially less efficacious, while the latter is more expensive and of unproven safety, but potentially more effective in the long-term [69–72]. A 2-year “add-on” treatment with LMV+ADV of 74 LMV-R HBeAg-negative patients resulted in no patient developing either a virological breakthrough or a genotypic resistance to ADV, enforcing the rationale for LMV+ADV combo therapy of LMV-R patients [73]. Combo therapy with LMV+ADV was demonstrated to be efficient in HBeAg-positive, HIV-confected patient developing LAM-R too, and in HBeAg-positive, genotype C infected, LMV-R patients in Japan [74,75]. Recently, an update to the original studies reported 90% rates of PCR negativity and <1% of cumulative incidence of ADV resistance in LMV-R patients receiving combo therapy [69,76,77]. Selection of timing for a rescue with ADV+LMV is of strategic importance in the management of LMV-R, as a complete virological response was achieved in all patients with baseline HBV DNA below 6 log_10_ cp/mL compared to only 75% of those with baseline viremia between 6 and 8 log_10_ cp/mL and 50% of those with higher than 8 log_10_ cp/mL viral load [73]. Early rescue prevented ALT reactivation too, compared to patients starting on therapy at higher levels of viremia who required more than 18 months of therapy to achieve disease remission [73]. Since hepatitis flares boost clinical decompensation in patients with cirrhosis, early “add-on” is mandatory in critically ill patients with LMV-R. The increased efficacy of early “add-on” rescue therapy, when compared with the late one, made of it now a recommended strategy for LMV-R patients, in order to also minimize the risk of multiple drug-resistant selection [78].

### 1.5. Prevention of Resistance

Resistance to anti-HBV analogues can be delayed or prevented by careful selection of patients to be treated, administration of *de novo* combination therapy, use of third generation NUCs as first line therapy and early adaptation of antiviral therapy, especially in partial responders. In the search for predictors of resistance to improve the cost-effectiveness of HBV therapy, a relationship between residual viral load at week 24 and risk of developing resistance at week 48 or 96, has been demonstrated [71]. According to the EASL Practice Guidelines, partial virological responders (PVR) to NUCs have a more than 1 log decline of viremia compared to baseline but still detectable serum levels of HBV DNA by real-time PCR assay (>10–15 U/ml) at week 24 or 48, depending on the genetic barrier of the anti-HBV drug [3].

The clinical relevance of NUCs PVR relates to the high risk these patients face of developing resistance to long-term anti-HBV treatment, particularly when first (LMV) and second generation (LdT, ADV) drugs are involved [3]. Conversely, for PVR to third generation NUCs, like ETV and TDF, though carrying a lower risk of resistance to long-term monotherapy, the association between residual viremia at week 48 and secondary treatment failure during follow-up has not been fully established [3]. Despite the strong rationale for adapting antiviral therapy, at least for selected NUCs, evidence-based algorithms for rescuing these patients have not been generated, apart from expert opinions.

Patients with undetectable HBV DNA at week 24 of LMV or LdT have a negligible (5%) risk of LMV-R in the following 18 months. LMV and LdT therefore should be continued as monotherapy only in those patients who achieve undetectable HBV DNA at week 24, provided that a regular HBV DNA monitoring is established to early identify the emergence of resistance and start an appropriate rescue strategy [39,79]. Patients with a detectable viremia at week 24 are at high risk of developing LMV and LdT resistance and, therefore, require early adaptation of antiviral therapy by either switching to a more potent antiviral agent or adding-on to another analog with a different resistance profile. Due to the lack of studies comparing these two strategies, either independently or head-to-head, an evidence-based indication cannot be offered on how patients with a PVR to LMV or LdT should be treated. To further reduce the yield of PVR at week 24, EASL Guidelines recommend LdT to be started as monotherapy in those patients with baseline viremia below 6 log_10_ IU/ml, only. For patients on ADV monotherapy, PVR at week 48 has been associated to a 50% chances of developing resistance in the following 3 years, suggesting, therefore, a rescue therapy for all PCR positive patients to prevent resistance [3]. *De novo* combination might represent the best approach for highly viremic patients in order to increase the antiviral efficacy while rescuing the risk of resistance, as suggested by studies in HIV patients. In general, the best strategy to prevent resistance is to start with potent and high genetic barrier drugs like ETV and TDF, which are expected to carry a negligible risk of drug resistance in clinical practice in the long term.

## 2. Treatment of Chronic Hepatitis C

Chronic hepatitis C is a major worldwide health problem with an estimated prevalence of 1.6–2% [80,81]. In Europe, more than 9 million chronic carriers and approximately 86,000 deaths per year are estimated due to the late complications of hepatitis C virus (HCV)[82]. The prognosis of chronic hepatitis C depends on the rate of fibrosis progression, which over a 20–30 year time span, may determine the risk of developing cirrhosis and its complications, namely HCC, liver decompensation, hepatic encephalopathy and oesophageal variceal bleeding [83]. The only therapeutic intervention able to halt this progressive process is eradication of HCV by Interferon (IFN)-based therapies.

**Figure 4.**
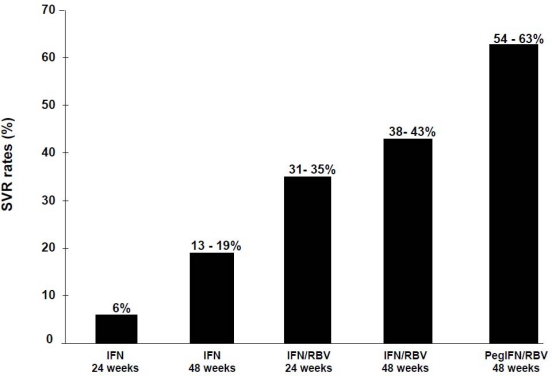
Attainable sustained virological response (SVR) rates to anti-HCV treatment.

Since the empirical choice to use IFN in 1986, therapy for chronic hepatitis C has constantly evolved over the past decade, with the attainable sustained virological response (SVR) rates increasing through the years [84] (Figure 4). The addition of the guanosine nucleoside analogue ribavirin (Rbv) to IFN can be considered the major breakthrough in the treatment of chronic hepatitis C [85,86]. Through mechanisms of action that still remain largely unknown [87], Rbv has determined a greater number of patients to ultimately achieve a SVR by increasing the rates of on-treatment response and reducing the rates of post-treatment relapse. In the large phase III clinical trials designed to assess its efficacy and safety, the combination of IFN and Rbv resulted in SVR rates of 30–35% in HCV genotype 1 patients and 75–80% in HCV-2 and 3 patients. These figures exceeded by far those obtained by IFN monotherapy, effectively leading the way for combination therapy to become the standard of care in the late 1990’s [85,86]. The latest innovation in the treatment of chronic hepatitis C has been the pegylation of the IFN molecule (PegIFN) through the attachment of one or more polyethylene glycols to the IFN, a process that is able to modify the immunological, pharmacokinetic and pharmacodynamic properties of the drug [88]. Standard IFN was in fact characterized by a number of limitations, such as poor stability, short elimination half-life and potential immunogenicity, that ultimately determined its small antiviral effect [89,90]. Moreover, due to the increase in elimination half-life obtained by the pegylation process, it has been possible to lengthen the dosing interval from the unpractical three times a week schedule required by standard IFN, to the more “user friendly” once a week administration, a feature that has increased convenience whilst facilitating adherence to the recommended treatment schedule. Following the demonstration of a more potent antiviral effect in terms of SVR rates in phase III randomized trials [91,92], PegIFN has become the standard of care for chronic hepatitis C. Currently, two forms of pegylated IFN exist: PegIFNα2a (Pegasys®; Hoffmann-LaRoche, Basel, Switzerland) and PegIFNα2b (PegIntron®; Schering-Plough, Kenilworth, NJ, USA), which show significant differences in terms of pharmacokinetics and dynamics, that ultimately might translate into different efficacy rates [93–99].

### 2.1. From registration trials to optimization studies

The superiority of combination therapy with PegIFN and Rbv was demonstrated by 2 international randomized multicenter studies [91,92]. In the PegIFNα2b trial by Manns *et al.*, the overall SVR rates achieved by the PegIFN/Rbv treatment arm were significantly higher than those obtained with standard IFN/Rbv (PegIFNα2b 54% *vs.* IFNα2b 47%), even if the benefit was restricted to patients infected by HCV genotype 1 (PegIFNα2b 42% *vs.* IFNα2b 33%). Quite surprisingly, in fact, this was not the case for patients with HCV genotypes 2 and 3 where the use of PegIFN did not translate into any apparent increase in SVR rates (PegIFNα2b 82% *vs.* IFNα2b 79%). On the contrary, in the PegIFNα2a trial by Fried *et al.*, PegIFNα2a/Rbv combination therapy resulted in significantly higher SVR rates in all HCV genotypes compared to standard IFN/Rbv (HCV-1: 46% *vs.* 36%, HCV-2 and 3: 76% *vs.* 61%). These data effectively led PegIFN plus Rbv combination therapy to supplant standard IFN as the standard of care treatment for naïve patients with chronic hepatitis C. Still, some limitations in the design of both studies required further multicenter trials, the so called optimization studies, to effectively determine the optimal dosing for each HCV genotype patient. In the study by Manns and colleagues, PegIFNα2b was administered coupled with a fixed dose of Rbv (800 mg/day), that clearly was underdosed, especially for patients with high body weight and HCV genotype 1. This was clearly shown by the Win-R study that demonstrated the superiority of the weight based dosing of Rbv over the fixed dose in patients with HCV genotype 1 [100]. Moreover, the Manns study could not assess the optimal treatment duration, since all patients were treated for 48 weeks independently from HCV genotype. The formal demonstration that patients with HCV-2 and HCV-3 can be successfully treated with a shorter course of combination therapy (24 weeks) was provided by the study by Zeuzem *et al.* in 2004. In that study, where HCV-2 and HCV-3 patients were treated for 24 weeks and compared with the patients treated for 48 weeks in the Manns study database, SVR rates were 79–93% in the 24 week treatment schedule and 88% for the 48 week historical control group [101]

For PegIFNα2a combination therapy, we have to thank the Hadziyannis study for providing us clinicians with a better individualized treatment schedule. In fact, the study clearly demonstrated that 48 weeks of treatment were better in terms of SVR rates than 24 weeks for patients with HCV-1, and that the higher dosing of Rbv, *i.e.* 1000 mg/day for those weighing less than 75 kg and 1200 mg/day for those weighing more than 75 kg, was associated with higher SVR rates than the fixed dose of Rbv (800 mg/day) (52% *vs.* 41%). On the contrary, the same study showed that for patients with HCV-2 or HCV-3, 24 weeks of combination therapy with a low dose of Rbv (800 mg) were sufficient to maximise treatment efficacy. Another noteworthy finding of the registration studies was the discovery of the so called HCV RNA week 12 stopping rule for HCV-1 patients. To that date, in fact, treatment was continued until week 24 in all patients, and therapy eventually discontinued in those who still tested serum positive for HCV RNA, the so called non responders. In the attempt to identify an earlier time point for stopping antiviral therapy, Fried and co-workers retrospectively identified in HCV RNA testing at week 12 the key time-point: a less than 2 Log decline in HCV RNA at this time point compared to baseline values was in fact highly predictive of a non-SVR, with only 3% of those patients ultimately achieving a SVR. This “stopping rule” was later validated also for PegIFNα2b [102], effectively leading the way for the study of early kinetics of HCV-RNA to become the standard of care in clinical practice in the years to follow [103]. Table 1 shows the recommended treatment schedules for naïve patients with chronic hepatitis C.

### 2.2. Towards Individualization of Antiviral Therapy

The Hadziyannis study should now be viewed as the first attempt to individualize anti-HCV therapy, anticipating the idea that SVR rates and tolerability can be maximized by customized schedules of treatment more than by a single “one for all” treatment regimen. Further efforts towards more refined individualized schedules were spurred by the growing evidence that the chances of achieving a SVR were related to the rapidity of serum HCV RNA clearance, SVR rates in fact being higher and relapse rates being lower in patients achieving HCV RNA negativity at week 4, the so called rapid virological response (RVR), than in any other subgroup of patients [104]. As a matter of fact, a small pilot study from Norway showed that patients infected with HCV-2 and HCV-3 who had achieved HCV-RNA undetectability at week 4 and 8 could successfully be treated with PegIFNα2b and Rbv given for just 14 weeks [105].

These findings coupled with the poor tolerability profile of PegIFN/Rbv combination therapy led researchers to analyze if shorter treatment periods could be an option for patients who achieved an RVR. The study by Zeuzem *et al.* in 2006 was the first to assess this matter in HCV-1 infected patients. 235 Patients with low baseline HCV-RNA values (<800,000 IU/mL) were treated with PegIFNα2b for 24 weeks and compared to an historical control arm, once again derived from the Manns study [106]. While the SVR rates were significantly lower in the 24 week treatment arm compared to the standard 48 week schedule (50% *vs.* 71%), if only patients with an RVR were analyzed, SVR rates did not differ between the 2 treatment groups (89% *vs.* 85%). Unfortunately, the study was not completely bias free as the primary end-point was not met and the efficacy data of the 24 week schedule in patients with an RVR derive from a subgroup analysis. Moreover, the lack of a true control arm in that study cannot be completely overlooked; still these data should be viewed as the first scientific proof that shorter treatment durations can be considered for this subset of patients. By retrospectively analyzing the data from the Hadziyannis study, Jensen and co-workers confirmed these findings also for the PegIFNα2a-based combination therapy in patients with an RVR. In fact, SVR rates were 89% in the 51 HCV-1 patients treated for 24 weeks and 77% in those treated for 48 weeks [107]. Altogether these data have led the EMEA to approve the 24 week treatment schedule of PegIFNα2a/2b plus Rbv for naïve HCV-1 patients with a low baseline viremia achieving an RVR.

The same paradigm was then also applied to patients with HCV-2 and HCV-3, a subgroup known to achieve SVR rates of approximately 70–80% with the standard of care 24 week schedule of PegIFN plus Rbv therapy. Multiple studies which addressed whether an abbreviated treatment regimen to 12–16 weeks could be proposed to the many HCV-2 and HCV-3 patients who achieved an RVR unfortunately differ significantly in terms of design, type of PegIFN utilized and Rbv dosing [108–111]. Taken altogether, these studies demonstrate that, first of all, an abbreviated treatment schedule should be proposed only to patients who achieve an RVR, as SVR rates are dismal if this end-point is not reached [110]. Secondly, what has consistently emerged is that while these abbreviated schedule strategies achieve high SVR rates, these are somewhat lower than those obtained by the standard 24 treatment schedules due to the higher relapse rate. The recently published non-inferiority study by Dalgard *et al.* further added to this, since while SVR rates in HCV-2 and HCV-3 patients with an RVR treated with PegIFNα2b/Rbv for 14 weeks were similar to those obtained with the standard 24 treatment course (81% vs 91%), the non-inferiority end-point was not met, effectively demonstrating that a shorter treatment course is inferior to the standard schedule [112]. Further working against an indiscriminate use of abbreviated therapies in patients with easy to cure HCV genotypes are the findings of a study in Italy showing increased risk of post-treatment relapse in patients with advanced fibrosis or high baseline body weight [113]. In conclusion, the jury on the efficacy of abbreviated therapies in HCV-2 and HCV-3 patients with an RVR is still out, the most reasonable approach being that abbreviated therapies should not be the standard of care for all HCV-2 and HCV-3 patients, but should only be considered in those who are intolerant to the standard 24 week duration [103].

Also unresolved is the issue regarding extended treatment duration in HCV-1 naïve patients with a delayed virologic response, defined as HCV RNA clearance between week 12 and 24 of treatment. These patients are characterized by high rates of post-treatment relapse, that were lowered by the extended treatment duration in at least 3 studies. In the Berg study the SVR rates in these patients were 29% in the 72 weeks extended treatment duration arm and 17% in the standard treatment group. The higher SVR rates were the direct consequence of lower relapse rates (40% *vs.* 64%) more than higher ETR rates (49% *vs.* 47%) [114]. The same was true for another study conducted utilizing PegIFNα2b 1.5 mcg/kg plus weight based Rbv in the same HCV-1 slowly responsive patients [115]. While these data suggest that if antiviral therapy is well-tolerated prolonged therapy to 72 weeks may be a viable option in this subset of patients [103], a recent multicenter randomized study showed no benefit of the extended treatment duration in HCV-1 slow responder patients [116]. In our opinion, these contrasting data coupled with the increase in terms of costs and side effects deriving from extending treatment duration to 72 weeks, suggest that this therapeutic strategy needs to be further validated before it can be implemented in the therapeutic algorithm of patients with chronic hepatitis C.

### 2.3. Retreatment of Persons Who Failed to Respond to a Previous Treatment

Roughly 20–50% of all treated patients will fail to achieve a SVR to IFN based therapies as a consequence of either primary non-response during treatment or post-treatment relapse following therapy discontinuation. In the wait for new more potent anti-HCV drugs that in theory should reduce the rates of treatment failure [117], effective strategies need to be developed for the retreatment of these patients, since most often treatment failure is observed in those who are most in need of anti-HCV treatment, *i.e.* those with advanced fibrosis. If the previous treatment consisted of standard IFN only, recent data show that the chance a SVR with PegIFNα2a or α2b plus Rbv is roughly 20% to 40% [118,119]. However, if the patient already received Rbv combined with either IFN or PegIFN, SVR rates are extremely low being close to 10% and 5%, respectively [118,119]. Due to the disappointing rates obtained, the recent AASLD guidelines do not recommend the re-treatment with PegIFN plus Rbv of these patients [103]. While this certainly is the most evidence based approach, there are patients that cannot wait 2–3 years to be retreated with the new direct anti-HCV drugs either due to an advanced disease or a sense of urgency to start a re-treatment. Recent data suggest that a 72 week treatment course of PegIFNα2a plus Rbv, both at standard dosage, might be the best therapeutic option in patients who have previously non responded to a course of PegIFNα2b plus Rbv [120]. The recently published REPEAT study, originally designed to assess the efficacy of 12 week 360 mcg/week PegIFNα2a plus weight based Rbv induction dose, followed by either 60 or 36 weeks of standard therapy, compared to the standard 180 mcg/week PegIFNα2a dose with weight based Rbv in patients who failed a previous PegIFNα2b plus Rbv course, showed that the patients treated for 72 weeks achieved significantly higher SVR rates compared to those who received 48 weeks (16% *vs.* 8%, p<0.001), with no apparent benefit from the 360 mcg/week PegIFNα2a induction dose. While the study enrolled too few patients with HCV genotypes 2 and 3 to draw any definite conclusions about the predictive role of HCV genotype on treatment outcome, bridging fibrosis/cirrhosis emerged once again as a strong predictor of treatment failure in the study. SVR rates in this subgroup of patients in fact were lower than 5%, effectively suggesting that the re-treatment of these patients should currently be deferred.

The study also demonstrated that antiviral treatment should be stopped in those patients who do not achieve HCV RNA negativity at week 12, since SVR in this subset was achieved in 4% of the patients independently from the treatment duration. While the overall observed 12% SVR rate is less than astounding, the demonstration of the superiority of the 72 week PegIFNα2a plus Ribavirin treatment arm led the EMEA to approve this regimen in previous non-responsive patients.

## 3. Conclusions

The management of chronic hepatitis B has evolved fast, several therapeutic options are now available and nowadays hepatitis B is a treatable disease. Therapy must reduce HBV DNA to as low a level as possible to ensure a degree of virological suppression that will then lead to biochemical remission, histological improvement and prevention of disease progression. IFN and NUC have both advantages and disadvantages, short-term treatment with Peg-IFN induces a sustained virological response in a third of patients, long-term NUCs treatment inhibits HBV replication in most of the patients, but drug resistance and safety in the long-term will remain the most important unresolved questions. Careful evaluation of patient history, staging of liver disease and virological factors should guide the start of treatment and the choice to the most appropriate treatment strategy. Antiviral therapy with third generation NUC such as TDF and ETV, early adaptation of therapeutic strategy in patients with partial virological response or treatment failure have significantly improved the long-term suppression rates, providing complete inhibition of HBV replication in the majority of patients for at least 5 years.

Currently, nearly 65% of all treated patients with chronic hepatitis C obtain persistent HCV viral eradication following PegIFN (α2a or α2b) plus Rbv combination therapy. While in the past years clinical research efforts have been geared toward individualizing treatment schedules on the basis of pre-treatment and on-treatment predictors of treatment outcome, the recent technical advances in cell culture systems and replication assays have finally provided researchers and pharmaceutical industries with the tools to develop and study new antiviral drugs in the treatment of chronic hepatitis C. These new drugs are particularly needed in patients who have failed to respond to a previous course of IFN plus Rbv, as the currently achievable SVR rates are far from being satisfactory. Many antiviral compounds have been evaluated for their efficacy in treating HCV patients specific protease and polymerase inhibitors are currently the most promising drugs being tested, with Telaprevir and Boceprevir recently entering Phase III studies. Both drugs have shown potent antiviral activity both *in vitro* and *in vivo*, however emerging as poor candidates for monotherapy due to the early emergence of drug resistant viral strains. This has forced researchers to focus on a triple therapy regimen, resulting from the combination of these drugs with PegIFN plus Rbv [121,122]. PegIFN and Rbv have in fact emerged as essential counterparts to reduce drug resistance and ultimately achieve persistent viral suppression, *de facto* reinforcing the concept that PegIFN and RBV will likely remain the backbone of antiviral therapy for years to come.

## Figures and Tables

**Table 1.** Recommended treatment schedules for naïve patients with chronic hepatitis C.

**Genotype**	**Peg-IFN dose**	**Daily Ribavirin Dose**	**Duration**	**Stopping Rule**
**HCV-1/4**	PegIFNα2a 180 μg/week	1000 mg if < 75 kg; 1200 mg if ≥ 75 kg	48 weeks	12° or 24° week if NR*
	PegIFNα2b 1.5 μg/kg/week	800 mg if < 65 kg; 1000 mg if ≥ 65 kg and ≤ 85 kg 1200 mg if > 85 kg and ≤ 105 kg ; 1400 mg if > 105 kg	48 weeks	12° or 24° week if NR*

**HCV-2/3**	PegIFNα2a 180 μg/week	800 mg	24 weeks	-
	PegIFNα2b 1.5 μg/kg/week	800 mg if < 65 kg; 1000 mg if ≥ 65 kg and ≤ 85 kg 1200 mg if > 85 kg and ≤ 105 kg; 1400 mg if > 105 kg	24 weeks	-

* NR (Non Response): less than 2 log HCV-RNA decline at week 12 or HCV-RNA detectability at week 24 of treatment
